# Beyond novice-to-expert progression: a context-enabled continuing professional development framework derived from nursing practice in resource-constrained South African hospitals

**DOI:** 10.3389/fmed.2026.1990761

**Published:** 2026-10-21

**Authors:** Maggie Nyelisani, Lufuno Makhado

**Affiliations:** 1 Department of Advanced Nursing Science, Faculty of Health Sciences, University of Venda, Thohoyandou, South Africa; 2 Department of Public Health, Faculty of Health Sciences, University of Venda, Thohoyandou, South Africa

**Keywords:** conceptual framework, continuing professional development, health workforce, nurses, professional competence, resource-constrained settings, South Africa, workplace learning

## Abstract

Continuing professional development (CPD) is frequently organised as a sequence of educational events directed at individual practitioners. In resource-constrained health services, however, participation and learning transfer are shaped by staffing, protected time, leadership, finance, infrastructure, access to current guidance and opportunities to apply learning. Individual-centred and linear developmental models do not adequately explain these interactions. This article proposes the Context-Enabled Continuing Professional Development (CE-CPD) Framework, derived from a programme of research involving a scoping review, qualitative evidence from professional nurses and nurse managers in Limpopo Province, South Africa, and critical engagement with developmental, implementation and CPD-outcomes literature. The framework positions professional learning within four interacting layers: a stakeholder and service ecosystem, organisational enabling conditions, a recurring five-phase workplace learning cycle, and an outcome pathway extending from access and participation to practitioner capability, professional behaviour, team and organisational performance, and patient or population health. Capability development is treated as domain-specific, iterative and potentially non-linear, rather than as an automatic progression from novice to expert. Six propositions specify how contextual readiness, protected opportunity, equitable access, workplace application, knowledge sharing and infrastructure are expected to influence these outcomes. The CE-CPD Framework shifts the explanatory focus from whether professionals attend CPD to whether their organisations make relevant learning possible and ensure its sustainable application. Although derived from nursing evidence, its organisational mechanisms may be relevant across healthcare professions. Interprofessional co-design, prospective testing and equity-sensitive evaluation are required before wider adoption.

## 1 Introduction

Continuing professional development (CPD) is intended to help healthcare professionals maintain and extend the capabilities required for safe, ethical and responsive practice throughout their careers. Its importance has increased as practitioners respond to changing population needs, new evidence, digital technologies, revised clinical guidance and more complex models of care. The World Health Organisation (WHO) accordingly places lifelong learning within the broader goal of building a competent and adaptable health workforce (1).

The educational logic underlying many CPD systems nevertheless remains narrow. Participation is often represented by attendance, hours or accumulated credits, while professional development is implicitly understood as an individual progression from limited knowledge towards expertise. Recent scholarship proposes a wider conception. Sklar et al. (2025) identify knowledge, skills, teams, quality improvement and prevention as interconnected CPD domains that should be aligned with healthcare value rather than treated as isolated educational objectives (2). Evidence linking CPD directly to patient outcomes remains promising but heterogeneous, partly because the pathway from an educational activity to clinical outcomes is complex and incompletely measured (3). Healthcare professionals also report that time, organisational support, resources and opportunities to apply learning determine whether education changes practice (4).

These limitations become especially visible in resource-constrained settings. A rapid review of CPD systems in low- and lower-middle-income countries found that leadership, stakeholder ownership, needs assessment, a context-responsive framework, financing, accreditation, appropriate learning materials and evaluation are required for sustainability (5). Evidence concerning regulated health professions further cautions that mandatory hours or credits should not be interpreted as direct measures of competence or improved care (6). In nursing, participation is similarly influenced by workload, staffing, managerial support, funding, access, relevance and career stage (7).

The wider African workforce context makes this problem consequential. Persistent shortages, maldistribution and uneven institutional capacity mean that the practitioners with the greatest need for accessible workplace learning may have the least opportunity to leave clinical services to attend it (8). In South Africa, the National Strategic Direction for Nursing and Midwifery Education and Practice calls for accessible, competency-oriented and digitally enabled CPD within supportive practice environments (9). The South African Nursing Council (SANC) framework positions CPD as a mechanism for maintaining professional standards (10), and the 2026 implementation circular confirms that the phased introduction of CPD for nurses and midwives is now in its third year (11).

Regulation alone does not explain how a facility with staff shortages, constrained budgets, limited connectivity and competing service demands can make professional learning achievable. Nor does it explain how learning is expected to move from an individual participant to colleagues, routine practice and patient care. A conceptual framework is therefore needed that connects the professional learning cycle to the organisational conditions under which learning is accessed and applied.

This article proposes the Context-Enabled Continuing Professional Development (CE-CPD) Framework. It was derived from nursing evidence generated in public hospitals in Limpopo Province, South Africa, and subsequently reconstructed as a testable model for resource-constrained health services. The framework does not present professional development as an automatic progression from novice to expert. It explains development as a recurring, context-dependent interaction between learning needs, workplace opportunities, organisational capacity, professional agency, feedback and service outcomes. The article has four objectives:

1. to explain the empirical and theoretical foundations of the CE-CPD Framework;
2. to specify its concepts and the proposed relationships among them;
3. to formulate propositions that can be tested through future implementation research; and
4. to consider its cautious adaptation to healthcare professions beyond nursing.

## 2 Why is an additional CPD framework needed

### 2.1 Distinguishing a conceptual framework from a theory

Theory, theoretical frameworks and conceptual frameworks should not be used interchangeably. A theory offers an explanatory system of concepts and relationships, while a theoretical framework describes how one or more theories are used to structure a study (12). A conceptual framework brings together concepts from empirical findings, existing theory and scholarly reasoning to provide an organised explanation of a phenomenon (12). It can therefore integrate several bodies of knowledge without claiming the predictive maturity of a fully tested theory.

Jabareen (2009) describes conceptual framework development as an iterative process of identifying, interpreting, organising and integrating concepts into a relational network (13). Consistent with this position, the CE-CPD Framework is presented as a provisional explanatory model. Its value depends on conceptual clarity, internal coherence, practical relevance and empirical testability. It should not be interpreted as evidence that the proposed relationships have already been causally demonstrated.

### 2.2 Limitations of an individual-centred interpretation of CPD

CPD systems commonly locate responsibility with the individual practitioner: the professional identifies a need, selects an activity, attends, reflects and records evidence (5, 6, 10). Professional agency is important, but this interpretation is incomplete where staff cannot obtain release time, activities are not locally available, internet access is unreliable, selection is inequitable or new practices cannot be implemented because equipment, guidance or managerial support is absent (4, 5, 7).

An exclusive focus on the learner can consequently misclassify a system constraint as poor motivation. A nurse who does not attend training while maintaining minimum staffing in a high-demand unit may be demonstrating professional responsibility rather than resistance to learning. Similarly, completion of an online module does not demonstrate that the learner had an opportunity to practise, receive feedback or alter care. The explanatory unit must therefore expand from the individual attendee to the interaction between practitioner, team, organisation, regulator, education provider and service environment.

### 2.3 Limitations of linear novice-to-expert progression

Benner's application of the Dreyfus model remains influential because it highlights experiential learning and shifts in professional judgement throughout the development of practice (14). However, the model can be overextended when CPD is represented as a simple mechanism that moves every practitioner from novice to expert. Expertise is domain-specific: an experienced nurse may be highly proficient in one clinical field and a beginner in another. Development may also be interrupted by changes in role, technology, disease patterns or clinical guidance. The CE-CPD Framework, therefore, uses developmental progression as a sensitising idea rather than a fixed ladder. It treats capability as dynamic and multidimensional. Knowledge, technical skill, judgement, teamwork, leadership and quality-improvement capability may develop at different rates. Progress depends on the quality of experience, supervision, feedback and opportunities for repeated application, not merely on the number of CPD activities completed.

## 3 Framework development and evidence base

### 3.1 Developmental approach

The framework was developed through an empirically informed conceptual-synthesis approach. This approach combined inductive concepts from a doctoral research programme with deductive concepts drawn from CPD, workplace learning and professional development literature. The framework-building process comprised four analytic operations:

1. Extracting recurrent concepts from the evidence base;
2. Grouping the concepts according to their function within a CPD system;
3. Mapping proposed relationships and feedback pathways; and
4. Examining the resulting framework for coherence, explanatory usefulness and testability.

The present article is not a secondary qualitative analysis intended to generate new participant themes. The previously reported empirical findings (15–17) are used as sources of concepts and contextual constraints. The new contribution is the organisation of those concepts into a relational model and the specification of testable propositions.

### 3.2 Empirical foundation

The first evidence stream was a scoping review conducted using the six-stage methodological framework of Arksey and O'Malley. The review examined literature published between January 2005 and October 2019 concerning professional nurses' experiences of participating in CPD in healthcare settings. Following title, abstract and full-text screening, 20 articles were included in the final synthesis. The findings were organised into four interrelated domains: professional and personal reasons for participating in CPD; barriers to CPD participation; the roles of nurse managers and human resource development managers; and organisational policies governing CPD. The review showed that CPD could strengthen professional knowledge, clinical competence, self-confidence, career development and quality of patient care. However, participation was constrained by financial limitations, staff shortages, heavy workloads, limited time, inadequate access to training and insufficient managerial support. These findings informed the framework's emphasis on structured CPD programmes, supportive workplace learning environments, adequate resources, equitable access to development opportunities and alignment between organisational policy, service needs and professional development (17).

The second evidence stream comprised qualitative interviews with 35 professional nurses who were purposively selected from six public hospitals in Limpopo Province: Tshilidzini and Elim hospitals in Vhembe District, Mankweng and Polokwane hospitals in Capricorn District, and Letaba and Nkhensani hospitals in Mopani District. Eligible participants had at least five years of professional nursing experience. Individual face-to-face, semi-structured interviews lasting approximately 30–45 min were conducted and analysed using Tesch's eight-step qualitative data analysis process. This shared dataset generated two complementary publications. The first examined professional nurses' experiences of CPD opportunities, including access to training, organisational support, resource constraints, staff shortages and proposed improvements (15). The second constituted a related re-analysis of the same underlying dataset, focusing on professional nurses' understanding and expectations of quality nursing care (16). Nyelisani et al. (15) and Nyelisani et al. (16), therefore, represent complementary analyses of a single cohort of professional-nurse participants and should not be interpreted as independent samples.

The third evidence stream comprised qualitative interviews with 10 nurse managers purposively drawn from the same six selected public hospitals in the Vhembe, Capricorn and Mopani districts. Eligible nurse managers had more than five years of experience in the selected public hospitals. Individual face-to-face, in-depth semi-structured interviews lasting approximately 30–45 min were audio-recorded, transcribed verbatim and analysed using Tesch's eight-step qualitative data-analysis process. The analysis generated three principal themes: nurse managers' understanding of CPD; their views of existing CPD activities in hospital settings; and proposed interventions to strengthen CPD. Nurse managers recognised CPD as encompassing personal and professional development, workplace-based training and knowledge sharing through post-training feedback. They identified inconsistent attendance, limited funding and resources, an inadequate number of specialist nurses, and concerns regarding staff selection processes as important barriers. Proposed improvements included management rounds to identify practice gaps, performance-development planning, staff recognition, systematic training-needs assessment and stronger coordination among nurse managers, human-resource-development personnel, CPD coordinators and the Department of Health (17).

### 3.3 Concept derivation and organisation

Across the three evidence streams, six recurrent problems were converted into framework requirements (15–17):

- a ***relevance gap***, where activities are insufficiently connected to individual, team or service needs;
- an ***opportunity gap***, where workload, staffing, distance or cost restricts participation;
- a ***capability-support gap***, where facilitators, mentors, equipment or current guidance are absent;
- an ***equity gap***, where scarce development opportunities are not distributed transparently;
- a ***learning-transfer gap***, where participants cannot apply or disseminate learning; and
- a ***feedback gap***, where participation is counted, but competence, behaviour and service change are not evaluated.

The concepts were then organised into four interacting layers: the stakeholder and service ecosystem, organisational enabling conditions, a recurring workplace learning cycle, and a progressive outcome pathway with an evidence-feedback loop. Outcome domains and feedback pathways were added to make the framework evaluable. Supplementary Table S1 documents the derivation of each principal CE-CPD construct, its empirical or theoretical source, its location within the framework, its relationship to the six propositions and its evidentiary status.

### 3.4 Ethical foundation and analytic integrity

The empirical programme was approved by the University of Venda Human and Clinical Trial Research Ethics Committee (SHS/20/PDC/17/1206). Permission was obtained from the Limpopo Provincial Department of Health and the relevant district and hospital authorities. Participants gave written informed consent. The present conceptual synthesis does not reproduce identifiable quotations or report new participant-level findings.

Analytic integrity was strengthened by tracing each principal framework construct to its empirical or theoretical source, as presented in Supplementary Table S1. Empirically observed conditions were distinguished from theoretically informed and newly proposed relationships. Because the framework has not undergone prospective implementation testing, all proposed causal pathways and moderating relationships are treated as hypotheses requiring prospective validation.

## 4 The context-enabled continuing professional development framework

### 4.1 Purpose and assumptions

The CE-CPD Framework explains how health professionals' learning may become accessible, relevant and transferable within resource-constrained services. It is based on five assumptions.

First, CPD is a recurring workplace process rather than a collection of unrelated events. Second, professional agency remains necessary but is insufficient without organisational opportunity and support. Third, capability development is domain-specific and non-linear. Fourth, meaningful CPD outcomes extend from participation to capability, behaviour, team practice, service performance and patient or population health. Fifth, evaluation findings should return to the next needs assessment and resource-allocation decision.

### 4.2 Stakeholder and service ecosystem

The outer structure of the framework represents the actors and service signals that shape CPD. These include healthcare professionals, peers, patients and communities, unit and facility managers, human resource development structures, quality assurance teams, district and provincial health departments, educational institutions, professional councils and other development partners.

Needs should not be defined by one actor alone. Individual practitioners can identify confidence gaps and career goals; peers and supervisors can identify teamwork or performance needs; service data can reveal guideline adherence or patient safety gaps; communities can indicate acceptability and access concerns; and regulators can define minimum professional requirements. Combining these perspectives reduces the risk that CPD is driven only by available courses, managerial preference or practitioner self-assessment and is consistent with evidence supporting multisource learning-needs assessment and attention to implementation context (18, 19).

### 4.3 Organisational enabling conditions

The middle structure comprises the conditions that determine whether the learning cycle can function:

1. ***governance and accountable leadership***, including defined responsibility, a facility plan, coordination and monitoring;
2. ***protected opportunity***, including safe staffing cover, scheduling and time to learn and apply learning;
3. ***equitable access***, including transparent selection and attention to shift, location, discipline and career stage;
4. ***human and educational support***, including prepared facilitators, specialist input, mentorship and peer learning;
5. ***financial, material and digital capacity***, including funding, venues, equipment, devices, connectivity and offline alternatives;
6. ***knowledge infrastructure***, including accessible and current policies, clinical guidelines and learning resources; and
7. ***a learning culture***, in which questioning, reflection, feedback and knowledge sharing are recognised as part of safe practice.

These enabling conditions synthesise the organisational barriers and facilitators identified in broader CPD literature and in the Limpopo empirical programme, particularly those concerning staffing, protected time, managerial support, funding, access, selection, learning resources and opportunities to apply learning (4, 5, 7, 15, 17).

These conditions operate as moderators rather than background details. A well-designed course may have a limited effect when staff cannot attend or apply it. Conversely, modest workplace learning may produce meaningful change when it is relevant, supported, repeated and connected to feedback.

### 4.4 The recurring workplace learning cycle

The CE-CPD cycle has five linked phases.

#### 4.4.1 Phase 1: identify and prioritise needs

Learning needs are identified by combining practitioner self-assessment with peer review, supervision, audit findings, evolving clinical guidance, population health priorities and service performance data. Needs assessment should distinguish a genuine competence gap from a performance problem caused principally by missing staff, equipment, medicines, authority or workflow. Learning-needs reviews indicate that combining several information sources is more defensible than relying on professional preference alone (18).

#### 4.4.2 Phase 2: co-plan learning and enabling support

Professionals, managers and educators jointly select objectives, delivery modes, resources, facilitators, protected time and indicators. The planning decision includes whether the need is best addressed through formal study, mentorship, simulation, case discussion, workplace demonstration, audit and feedback, digital learning or an interprofessional activity. Equity is addressed prospectively through explicit selection and access criteria. The feasibility of the selected approach must therefore be considered alongside the organisational conditions required for participation and implementation (4, 5, 15, 17).

#### 4.4.3 Phase 3: learn and apply in practice

Learning is enacted through formats suited to the competency and context. Knowledge acquisition may occur through short courses or online resources, while procedural and relational competencies require demonstration, supervised practice and feedback. Application is included in this phase because the transfer of learning cannot be assumed to occur after an activity ends. Workplace application, supervision and feedback are particularly important where CPD is expected to influence professional behaviour rather than knowledge alone (4, 7, 15, 17).

#### 4.4.4 Phase 4: reflect, assess and evaluate

Practitioners consider what changed, what remains uncertain and which contextual barriers affected the application. Assessment may include observation, simulation, case review, audit or reflective evidence. At the organisational level, evaluation examines reach, equity, competence, practice behaviour, team functioning and selected service indicators rather than attendance alone. This multilevel evaluation approach is necessary because participation and knowledge acquisition cannot be treated as equivalent to practice or patient-level change (3, 5).

#### 4.4.5 Phase 5: share, adapt and reinvest

Learning is distributed through peer teaching, case review, mentoring, updated local procedures or quality-improvement work. Evaluation findings are used to adapt the intervention, correct implementation barriers and identify the next learning priority. This phase turns individual participation into a potential resource for the team and organisation. Structured feedback and peer dissemination address the learning-transfer problems identified in both the broader literature and the evidence from Limpopo (4, 15, 17).

### 4.5 Developmental trajectories

Benner's developmental perspective (14) informs the framework's attention to experience, judgement and situated learning. The CE-CPD Framework nevertheless replaces a single novice-to-expert line with multiple capability trajectories. A practitioner's position varies by clinical domain, role and task. Movement can be forward, stable or temporarily backward when practice changes. The relevant question is not whether a professional has become a globally recognised expert, but whether capability in a defined area has developed sufficiently for safe, context-appropriate performance and whether that capability is sustained.

### 4.6 Outcomes and feedback

The outcome pathway combines implementation-context reasoning from the updated Consolidated Framework for Implementation Research (19) with Moore et al.'s distinction among participation, learning, competence, performance and health outcomes (20). CE-CPD extends these contributions by incorporating equity, team and organisational effects and a feedback loop into the subsequent learning cycle:

- ***access and participation:*** who was reached, excluded, retained or unable to participate;
- ***learning and capability:*** knowledge, skill, judgement, confidence and preparedness;
- ***professional behaviour:*** application, adherence, communication and reflective practice;
- ***team and organisational performance:*** knowledge diffusion, coordination, workflow and quality improvement; and
- ***patient, service or population outcomes:*** safety, experience, clinical processes, access, equity and health outcomes.

The pathway should be interpreted as a sequence of contributions rather than a direct causal chain. Contextual determinants influence every stage, and more distal patient, service or population outcomes depend on successful changes in capability, professional behaviour and team or organisational performance. The framework, therefore, does not claim that a single educational activity directly causes a patient or population outcome.

Figure 1 summarises the four-layer architecture, proposed relationships and feedback pathway. Table 1 translates the principal framework constructs into operational questions and illustrative measures for future evaluation.

**Figure 1:**
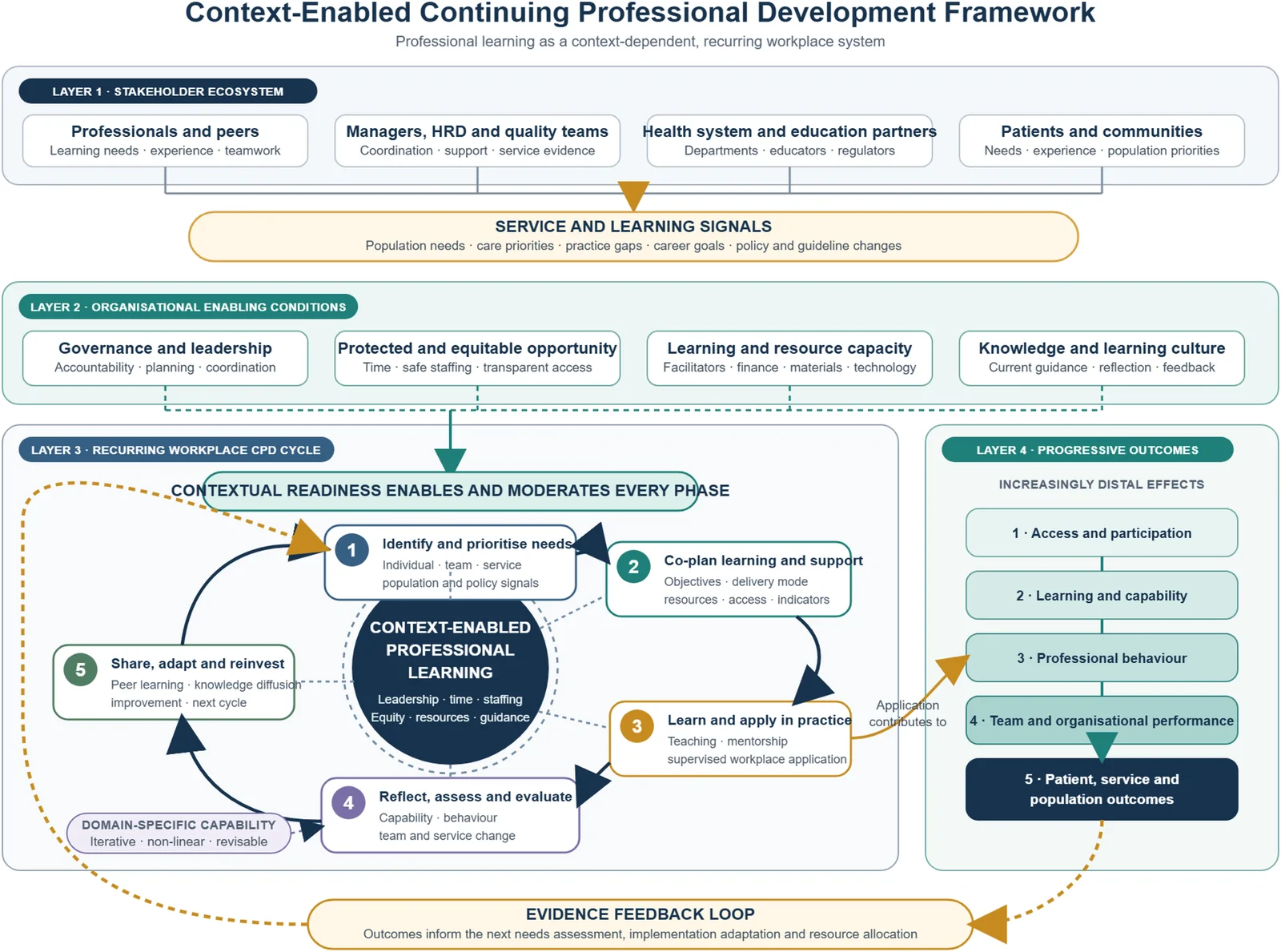
Context-enabled continuing professional development framework. The framework conceptualises CPD as a recurring workplace system comprising four interacting layers: the stakeholder and service ecosystem, seven organisational enabling conditions, a five-phase workplace learning cycle and progressively distal outcomes. Contextual readiness influences each learning phase, while the evidence-feedback pathway informs subsequent learning-needs assessment and resource-allocation decisions. Capability development is iterative, domain-specific and potentially non-linear. Empirically informed components are distinguished from theoretically proposed relationships requiring prospective validation.

**Table 1:** CE-CPD constructs and illustrative measures.

Construct	Operational question	Illustrative measures
Stakeholder participation and needs alignment	Is CPD directed at a demonstrable practitioner, team, service, or population need?	Source of identified need; stakeholder groups involved; alignment with audit findings, clinical guidance or service priorities
Governance and accountable leadership	Are responsibility, coordination and accountability for CPD clearly defined?	Approved facility plan; designated CPD lead; implementation reports; management-review frequency
Protected opportunity	Can staff participate in and apply learning safely within service demands?	Protected learning hours; staffing cover; cancellation rate; workload; opportunities for supervised application
Equitable access	Are development opportunities distributed transparently across staff groups and settings?	Participation by facility, unit, shift, profession, career stage and remoteness; selection criteria; reasons for exclusion
Human and educational support	Are appropriate facilitation, mentorship, specialist input and feedback available?	Facilitator preparation; mentorship contacts; supervised-practice episodes; feedback completion
Financial, material and digital capacity	Are the resources required for learning and workplace application available?	CPD budget; venue availability; equipment functionality; connectivity; device access; technical support; offline alternatives
Knowledge infrastructure	Can staff access current guidance and learning resources at the point of care?	Guideline currency; accessibility; staff orientation; use of guidance during audit, supervision or case review
Learning culture	Are reflection, questioning, feedback and knowledge sharing supported?	Psychological-safety measures; reflective sessions; peer-review participation; leadership response to identified learning needs
Workplace application and learning transfer	Is learning demonstrated and applied in routine practice?	Observed behaviour; simulation or competency assessment; case documentation; audit findings; practice changes
Reflection, assessment and evaluation	Are learning, behaviour and contextual barriers assessed beyond attendance?	Knowledge and skill assessment; reflective evidence; behaviour indicators; reach, feasibility, acceptability and equity measures
Knowledge sharing and adaptation	Is individual learning shared, adapted and used by the wider team?	Peer teaching; mentoring; case review; updated local procedures; quality-improvement activities; adaptation records
Outcomes and feedback	Do evaluation findings inform the next learning and resource-allocation cycle?	Practitioner capability; professional behaviour; team and service indicators; patient outcomes; completed improvement actions

## 5 Testable propositions

The framework generates six propositions for empirical examination.

***Proposition 1: contextual readiness proposition.*** The association between perceived learning need and CPD participation will be stronger where facilities provide accountable leadership, protected time and adequate learning resources.
***Proposition 2: workplace-application proposition.*** Improvement in professional behaviour will depend more strongly on opportunities for supervised workplace application and feedback than on attendance or knowledge gain alone.
***Proposition 3: equity proposition.*** Transparent selection, decentralised delivery and monitoring of participation across staff groups will reduce unequal access and the concentration of specialist capability.
***Proposition 4: knowledge-diffusion proposition.*** CPD activities that include structured peer sharing, mentorship or team-based quality improvement will produce greater organisational effects than activities ending with individual completion.
***Proposition 5: digital-conditionality proposition.*** Digital CPD will improve reach only when connectivity, devices, digital capability, protected time, technical support and accessible offline alternatives are sufficiently available.
***Proposition 6: mediated-outcomes proposition.*** Effects on patient or population outcomes will be mediated by changes in capability, professional behaviour and team or organisational performance and moderated by the availability of the resources needed to implement new practice.

## 6 Discussion

The CE-CPD Framework changes the central CPD question from “Why did the professional not attend?” to “What combination of need, opportunity, support, application and feedback makes professional learning possible and useful here?” This shift is important in resource-constrained environments because it prevents organisational failures from being attributed automatically to individual motivation.

The framework also connects three areas often treated separately: professional capability development, implementation context and outcome evaluation. Developmental models draw attention to experience and judgement; implementation frameworks draw attention to context; and CPD-outcomes models distinguish learning from behaviour and health outcomes. The CE-CPD Framework integrates these contributions into a recurring workplace cycle, grounded in the realities reported by nurses and managers in Limpopo (15–17).

The framework complements Sklar and colleagues' five CPD domains of knowledge, skills, teams, quality improvement and prevention (2). Those domains clarify what CPD may seek to develop. CE-CPD focuses on the contextual mechanisms through which such development becomes possible, equitable and transferable. It also aligns with evidence from low- and lower-middle-income settings that sustainable systems require leadership, stakeholder ownership, a guiding framework, financing, needs assessment, accreditation and evaluation (5).

Its distinctive contribution is to place enabling conditions around every phase of the learning cycle rather than listing context as an external barrier. Staffing, protected time and resources are causal assumptions within the model: they influence whether needs are identified accurately, plans are feasible, learning is accessed, application occurs, and evaluation is used.

The original framework used Benner's novice-to-expert model (14) to describe the progression from novice to expert practice. The revised framework retains the importance of situated experience but rejects two problematic simplifications. First, registered professionals should not be categorised globally as novices merely because they require CPD. Second, expertise should not be inferred from participation.

Career-stage evidence supports differentiated learning needs: early-career practitioners may require consolidation and supervision, whereas experienced practitioners may need specialist, educator, leadership or research development (21). The same practitioner can nevertheless occupy different developmental positions across competencies. CE-CPD therefore uses capability profiles rather than global labels and requires direct assessment where competence claims are made.

Digital delivery is attractive where geography and staffing limit attendance. Health-facility managers in Rwanda identified institutional strategy, infrastructure, technical support, digital capability and leadership as determinants of online CPD adoption (22). WHO similarly cautions that digital education can reproduce inequity when connectivity, devices, support or digital literacy are uneven (23).

The CE-CPD Framework, therefore, does not position technology as a separate solution. It locates digital delivery within the enabling context and requires low-bandwidth, mobile-compatible and offline alternatives. Digital participation should also be assessed by location, shift, profession and access to equipment. Practical competencies continue to require opportunities for demonstration, observed performance and feedback.

South Africa's phased CPD implementation creates an opportunity to strengthen lifelong learning, but a credit-focused response could reproduce an attendance model (10, 11). Regulation can set expectations and support accountability; employers must still provide viable access and application conditions. Professionals retain responsibility for reflective and ethical practice, while facilities and health-system structures hold corresponding responsibilities for time, staffing, resources, fair access and learning support.

This reciprocal account avoids two extremes. CPD should neither be reduced to personal compliance nor treated as something for which practitioners have no responsibility. The framework proposes distributed accountability in which practitioner agency and organisational capacity are both necessary.

The framework was derived from nursing and should be described transparently as such. Its mechanisms may nevertheless apply to medicine, pharmacy, rehabilitation, emergency care, laboratory services and other health professions, as all may experience constraints related to staffing, time, funding, geography, infrastructure and learning transfer.

Interprofessional application requires more than relabelling “nurse” as “healthcare professional”. Professional councils, scopes of practice, competency standards and learning traditions differ. Adaptation should involve representatives of relevant professions, managers, educators, regulators and service users. Common constructs (such as needs alignment, protected opportunity, equitable access, team learning and outcome feedback) can be retained while profession-specific competencies and assessment methods are co-designed.

## 7 Evaluation and research agenda

The CE-CPD Framework should be tested as a programme theory rather than adopted as a validated intervention. An initial study could assess content validity and contextual relevance through interprofessional Delphi or nominal group processes that include frontline professionals, managers, regulators, educators, finance personnel, digital support personnel and patient representatives.

A subsequent mixed-methods pilot could compare facilities with different levels of contextual readiness. Measures should examine reach, acceptability, feasibility, equity, capability, workplace behaviour, knowledge diffusion, service indicators and cost. The economic dimension is important because CPD evaluations rarely consistently capture staff release time, delivery costs and opportunity costs (24). A longer follow-up is needed to determine whether learning and practice changes are sustained.

Testing should examine both mechanisms and outcomes. For example, a study of digital CPD should measure whether connectivity and protected time moderate completion, while a mentorship intervention should examine whether feedback mediates behaviour change. Equity-sensitive analyses should disaggregate access and outcomes by facility, remoteness, unit, shift, profession, career stage and other locally relevant dimensions.

## 8 Strengths and limitations

The framework is grounded in the experiences of frontline professional nurses, managerial perspectives and a broader synthesis of evidence. It addresses context explicitly, specifies the relationships it proposes and provides measurable constructs and hypotheses. It also distinguishes participation, capability, behaviour, organisational performance and patient outcomes.

The framework has important limitations. Its empirical foundation is drawn from public hospitals in one South African province and is dominated by nursing evidence. Private hospitals, primary healthcare settings, other health professions, patients and community representatives were not involved in its original development. The manuscript synthesises previously reported evidence rather than analysing a new sample of participants. The framework has not undergone prospective implementation testing or interprofessional validation; therefore, its pathways and propositions represent theoretically plausible relationships rather than established causal effects.

The original scoping review covered literature published up to October 2019, although more recent literature was incorporated into the present conceptual article to refine and contextualise the framework. Wider political, labour-market, and fiscal determinants operating beyond the facility's control may also be insufficiently represented.

## 9 Conclusion

Professional learning in resource-constrained health services cannot be explained adequately as an individual journey from novice to expert. It is shaped by whether relevant needs are identified, learning is co-planned, staff have protected opportunities to participate, resources and guidance are available, new practices can be applied, and learning is shared and evaluated. The CE-CPD Framework positions these conditions within a recurring workplace learning system. It retains professional agency and experiential development while making organisational responsibility, equity, implementation context and outcome feedback explicit. The framework offers six testable propositions and a practical structure for future co-design and evaluation. Its broader value will depend on prospective testing, economic and equity analysis, and validation with other healthcare professions and service users.

## Data Availability

The original contributions presented in the study are included in the article/Supplementary Material, further inquiries can be directed to the corresponding author.
