# Supplementary material for: Beyond novice-to-expert progression: a context-enabled continuing professional development framework derived from nursing practice in resource-constrained South African hospitals
Source: Front Med (Lausanne). 2026 Oct 21;13:1990761. doi: 10.3389/fmed.2026.1990761 (PMC13730842; doi:10.3389/fmed.2026.1990761)
Supplement: Supplementary file 1 [file Table1.docx]

**Supplementary Table S1. Construct derivation and evidentiary status**

| **Source finding or theory** | **Extracted problem or insight** | **CE-CPD construct** | **Framework location** | **Proposition** | **Evidentiary status** |
| --- | --- | --- | --- | --- | --- |
| CPD needs may not align with practitioner or service priorities (5, 17) | Relevance gap | Needs alignment | Ecosystem and Phase 1 | P1 | Empirically observed and theoretically informed |
| Staffing shortages, workload and time restrict participation (7, 15, 17) | Opportunity gap | Protected opportunity | Organisational enabling conditions | P1 | Empirically observed |
| Staff-selection processes may be unclear or inequitable (15, 17) | Equity gap | Equitable access | Enabling conditions and Phase 2 | P3 | Empirically observed |
| Limited managerial coordination weakens CPD implementation (5, 15, 17) | Governance gap | Accountable leadership | Organisational enabling conditions | P1 | Empirically observed and theoretically informed |
| Mentorship, specialist support and feedback may be unavailable (4, 7, 17) | Capability-support gap | Human and educational support | Enabling conditions and Phase 3 | P2 | Empirically observed |
| Funding, equipment and infrastructure constrain learning and application (5, 15, 17) | Resource gap | Financial, material and digital capacity | Organisational enabling conditions | P1 and P5 | Empirically observed |
| Current guidance may not be available at the point of care (4, 5) | Knowledge-access gap | Knowledge infrastructure | Organisational enabling conditions | P1 and P2 | Theoretically informed |
| Attendance may occur without opportunities for practice application (4, 15) | Learning-transfer gap | Workplace application | Phase 3 | P2 | Empirically observed |
| Post-training feedback and dissemination are inconsistent (15, 17) | Knowledge-diffusion gap | Peer sharing and knowledge diffusion | Phase 5 | P4 | Empirically observed |
| Participation is frequently measured without behaviour or service outcomes (3, 5, 20) | Evaluation gap | Multilevel evaluation | Phase 4 and outcomes pathway | P6 | Empirically and theoretically informed |
| Digital learning may increase reach while reproducing resource inequalities (22, 23) | Digital conditionality | Digital readiness | Enabling conditions | P5 | Theoretically informed; requires prospective validation |
| Expertise varies by domain and may not progress linearly (14, 21) | Limitations of global novice-to-expert classification | Multiple capability trajectories | Section 4.5 | — | Theoretically informed |
| Multiple actors generate different learning signals (5, 17, 19) | A single-source needs assessment is insufficient | Stakeholder ecosystem | Ecosystem and Phase 1 | P1 and P3 | Theoretically informed |
| Evaluation should influence later learning and allocation decisions (5, 20) | Weak feedback between outcomes and future planning | Evidence-feedback loop | Phase 5 and outcome pathway | P6 | The newly proposed integration requires validation |
| Patient outcomes depend on intermediate capability and behaviour changes (3, 20) | Direct educational causation is implausible | Mediated outcome pathway | Section 4.6 | P6 | Theoretically proposed; requires prospective validation |
| Organisational readiness influences every learning phase (5, 19) | Context cannot be treated as an external background variable | Contextual readiness | Cross-cutting moderator | P1, P5 and P6 | The newly proposed integration requires validation |

Note: *Reference numbers correspond to the reference list in the main manuscript.*
